# Calcification of thyroid nodules increases shear-wave speed (SWS) measurement: using multiple calcification-specific SWS cutoff values outperforms a single uniform cutoff value in diagnosing malignant thyroid nodules

**DOI:** 10.18632/oncotarget.11710

**Published:** 2016-08-31

**Authors:** Bao-Ding Chen, Hui-Xiong Xu, Yi-Feng Zhang, Bo-Ji Liu, Le-Hang Guo, Dan-Dan Li, Chong-Ke Zhao, Xiao-Long Li, Dan Wang, Shuang-Shuang Zhao

**Affiliations:** ^1^ Department of Medical Ultrasound, Shanghai Tenth People's Hospital, Ultrasound Research and Educational Institute, Tongji University School of Medicine, Shanghai, China; ^2^ Department of Medical Ultrasound, Shanghai Tenth People's Hospital, Clinical College of Nanjing Medical University, Shanghai, China; ^3^ Thyroid Institute, Tongji University School of Medicine, Shanghai, China; ^4^ Shanghai Center of Thyroid Diseases, Shanghai, China; ^5^ Department of Medical Ultrasound, Affiliated Hospital of Jiangsu University, Zhenjiang, China

**Keywords:** thyroid nodule, calcification, ultrasound, shear-wave elastography, point shear-wave measurement

## Abstract

Conventional ultrasound cannot satisfactorily distinguish malignant and benign thyroid nodules. Shear-wave elastography (SWE) can evaluate tissue stiffness and complement conventional ultrasound in diagnosing malignant nodules. However, calcification of nodules may affect the results of SWE. The purposes of this study are to compare the differences of shear-wave speed (SWS) measurement among different calcification groups and compare the diagnostic performance between using a single uniform SWS cutoff value and multiple individual calcification-specific cutoff values using technique of point SWS measurement. We retrospectively identified 517 thyroid nodules (346 benign and 171 malignant nodules) examined by conventional ultrasound and point SWS measurement. There were 177 non-calcified, 159 micro-calcified and 181 macro-calcified nodules. The diagnostic performance was evaluated by receiver operating characteristic (ROC) curve and area under the curve (AUC) was computed. The mean SWS in malignant nodules more than doubled that of benign nodules (4.81±2.03 m/s vs. 2.29±0.99 m/s, p<0.001). The mean SWS of nodules progressively increased from the non-calcification (2.60±1.49 m/s), to micro-calcification (3.27±1.85 m/s) and to macro-calcification (3.68±2.26 m/s) groups (p<0.001), which was true in both the benign and malignant nodules. If we used individual SWS cutoff values for non- (SWS >2.42 m/s), micro- (SWS >2.88 m/s) and macro-calcification (SWS >3.59 m/s) nodules in the whole group, the AUC was 0.859 (95% confidence interval [CI], 0.826-0.888), which was significantly better than the AUC of 0.816 (95% CI, 0.780-0.848) if a single uniform cutoff value (SWS >2.72 m/s) was applied to all the nodules regardless of calcification status (p=0.011). The cutoff values of SWS for different calcified nodules warrant future prospective validation.

## INTRODUCTION

The incidences of thyroid nodules and thyroid cancer are increasing worldwide, largely due to enhanced diagnostic practices [1-4]. China has by far the largest population in the world and the burden of clinical management of thyroid nodules and thyroid cancer is enormous. A recent large community-based study revealed a thyroid nodule prevalence of 49% in Chinese adults by conventional ultrasound examination [3]. Nevertheless, only a small percentage of thyroid nodules are malignant [5]. It is critical to differentiate malignant from benign thyroid nodules and avoid unnecessary fine needle aspiration (FNA) biopsy and surgery. Conventional B-mode (brightness mode, or 2D mode) ultrasound is the most commonly used method to detect and evaluate thyroid nodules. Conventional ultrasound imaging characteristics associated with malignant nodules include the presence of micro-calcifications, hypoechogenicity, size greater than 2 cm, taller-than wide shape, and an entirely solid composition [6], but these features have varing sensitivity and specificity for diagnosing malignant thyroid nodules [7].

In recent decade, there have been many studies evaluating whether elastography, a non-invasive ultrasound method to measure tissue stiffness, can complement conventional ultrasound in differentiating malignant from benign nodules [8]. Conventional ultrasound images reveal differences in the acoustic properties of soft tissues, whereas elastography is able to reveal the differences in the elastic properties of soft tissues [9]. It is known that cancer tissues are stiffer than normal tissues [8]. There are two types of elastography, strain elastography (SE) and shear-wave elastography (SWE). Conventional SE requires manual compression by the operator, which can only produce semi-quantitative images and cannot precisely measure tissue stiffness. In contrast, SWE includes shear-wave speed (SWS) imaging and point shear-wave speed (pSWS) measurement, which can evaluate the tissue stiffness qualitatively and quantitatively by monitoring the SWS propagation in tissues [9, 10]. A number of studies [11-14] have shown that SWE is a promising complementary ultrasound technique for differentiating malignant and benign thyroid nodules.

Several factors can affect the results of elastography, particularly calcification in nodules [15-17]. Veyrieres et al. [17] reported that calcification in thyroid nodules increased SWS values on pSWS measurement, thus resulting in higher number of false positive nodules when a uniform cutoff value of SWS was applied for non-calcified and calcified nodules. Some prior studies simply excluded calcified nodules from their analysis to avoid this problem [18]. However, excluding calcified nodules in SWS analysis may miss a significant number of true malignant nodules, because calcified nodules account for 19.8-38.6% of all thyroid nodules [19, 20] and carry a doubled risk of being malignant nodules compared to non-calcified nodules [21-24].

The purposes of this study were to compare the differences of SWS on pSWS measurement among nodules with different calcifications (non-, micro-, and macro-calcification) and to compare the diagnostic performance between a single cutoff value and individually defined cutoff values of SWS in diagnosing malignant thyroid nodules.

## RESULTS

### Basic characteristics of patients and nodules

There were 517 thyroid nodules in 498 patients, including 346 (66.3%) benign and 171 (33.7%) malignant nodules. Among 498 patients, 107 were men and 391 were women, and the mean age (± SD) of patients was 51.5 ± 12.9 years (range, 18-82 years). Based on the pattern of calcification in nodule, these thyroid nodules were classfied into non-calcification (n=177), micro-calcification (n=159) and macro-calcification groups (n=181). There were 140 benign and 37 malignant non-calcified nodules, 94 benign and 65 malignant micro-calcified nodules, and 112 benign and 69 malignant macro-calcified nodules. The mean size (±SD) of nodules was 20.1 ± 9.2 mm, ranging from 10.0 to 62.6 mm. There were statistically significant differences in terms of patient age, calcified pattern of nodule, size of nodule, maximum SWS of nodule, and mean SWS of nodule between the benign and malignant groups (Table 1).

**Table 1:** Basic characteristics of patients and nodules

Characteristics	Whole group * (n=517)	Benign group * (n=346)	Malignant group * (n=171)	*P* value @
No. of patients	498	330	168	
Age (yr) #	51.5 ± 12.9 [18-82]	52.6 ± 13.2 [18-82]	49.3 ± 12.3 [23-78]	0.006
Males/females	107/391	78/253	29/138	0.127
Pattern of nodule				<0.001
Non-calcification	177 (34.2)	140 (40.5)	37 (21.7)	
Micro-calcification	159 (30.8)	94 (27.2)	65 (38.0)	
Macro-calcification	181 (35.0)	112 (32.3)	69 (40.3)	
Size (mm) #	20.1 ± 9.2 [10.0-62.6]	21.3 ± 10.1 [10.0-62.6]	17.6 ± 6.5 [10.0-39.2]	<0.001
No. of 10-20 mm	363 (70.2)	220 (63.6)	143 (83.6)	
No. of >20 mm	154 (29.8)	126 (36.4)	28 (16.4)	
Maximum SWS of nodule # (m/s)	4.24 ± 2.55 [0.54-8.40]	3.47 ± 2.16 [0.54-8.40]	5.81 ± 2.56 [1.17-8.40]	<0.001
Mean SWS of nodule # (m/s)	3.12 ± 1.84 [0.35-8.40]	2.29 ± 0.99 [0.35-6.12]	4.81 ± 2.03 [1.42-8.40]	<0.001
Maximum SWS of surrounding tissue # (m/s)	2.60 ± 0.80 [0.82-7.64]	2.56 ± 0.92 [0.82-7.64]	2.56 ± 0.73 [0.90-5.88]	0.095
Mean SWS of surrounding tissue # (m/s)	2.28 ± 0.74 [0.42-6.24]	2.25 ± 0.76 [0.66-6.24]	2.31 ± 0.69 [0.42-5.34]	0.420

* Numbers in parentheses are percentages.

# Data are means ± standard deviations. Ranges are in brackets.

@ *P* values of nonparametric variables were determined by Chi-square test, and *P* values of continuous variables by independent t test.

Abbreviation: SWS, shear-wave speed.

### Features of nodules among different calcification groups

**Figure 1:**
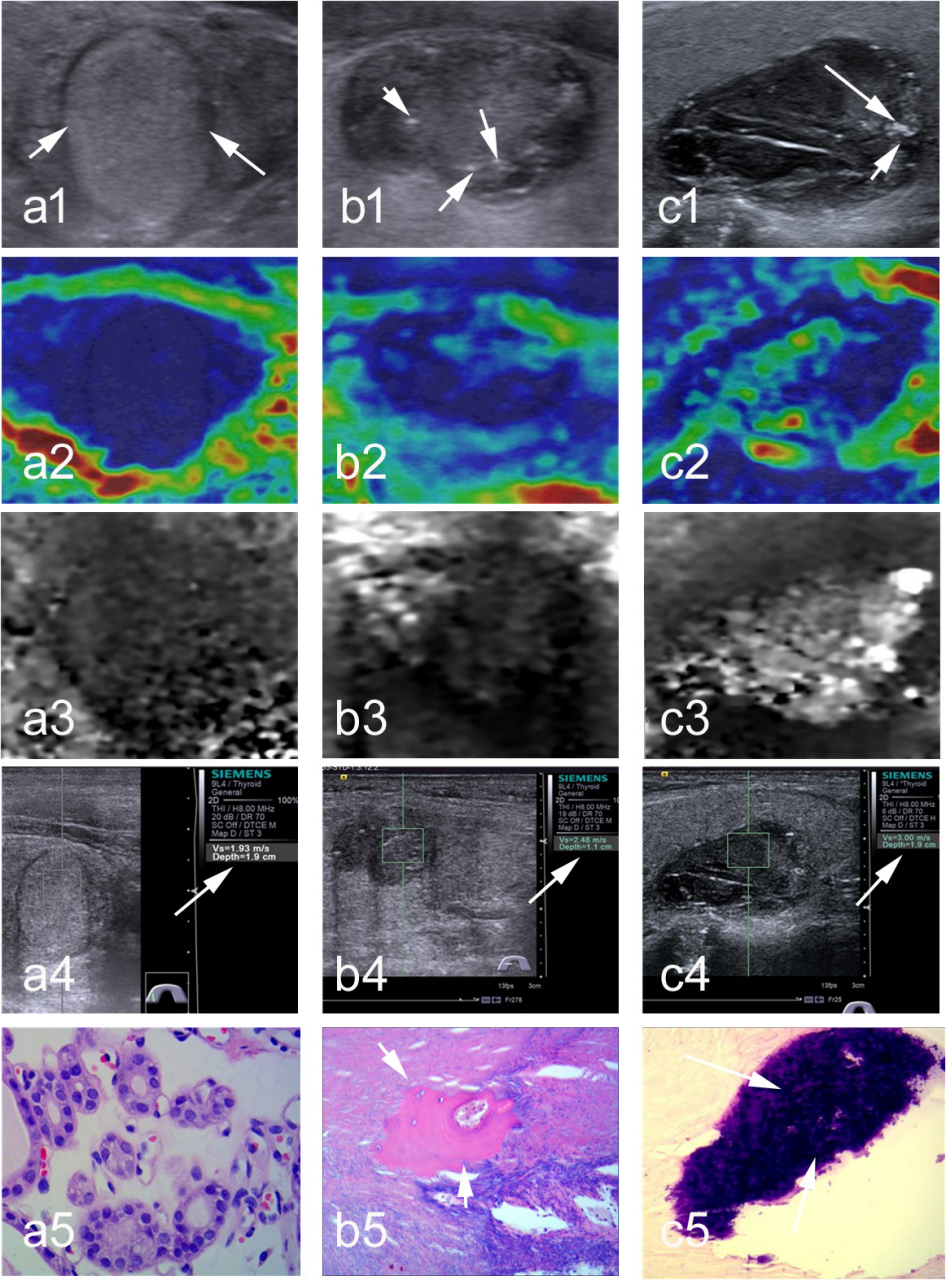
Representative images and histology of non-calcified, micro-calcified and macro-calcified benign thyroid nodules (a1) At conventional ultrasound, a non-calcified nodule is 16 mm in dimension, solid, isoechogenic, well defined, taller than wide; (a2-a3) at SE, conventional SE score is 4, and ARFI SE grade is 3; (a4) at SWE, the SWS of 1.96 m/s in nodule is assigned (arrow); (a5) Surgery histology confirms a follicular thyroid adenoma (×400). (b1) At conventional ultrasound, a micro-calcified (arrows) nodule is 14 mm in dimension, solid, hypoechogenic, poorly defined, wider than tall; (b2-b3) at SE, conventional SE score is 3, and ARFI SE grade is 3; (b4) at SWE, SWS of 2.48 m/s in nodule is assigned (arrow); (b5) Surgery histology confirms the Hashimoto's nodule with micro-calcified foci (arrow, ×400). (c1) At conventional ultrasound, a macro-calcified (arrows) nodule is 21-mm in dimension, solid, hypoechogenic, well defined, taller than wide; (c2-c3)at SE, conventional SE score is 2, ARFI SE grade is 2; (c4) at SWE, SWS of 3.00 m/s in nodule is assigned; (c5) Surgery histology confirms a nodular goiter with macro-calcified foci (arrow, ×100).

**Figure 2:**
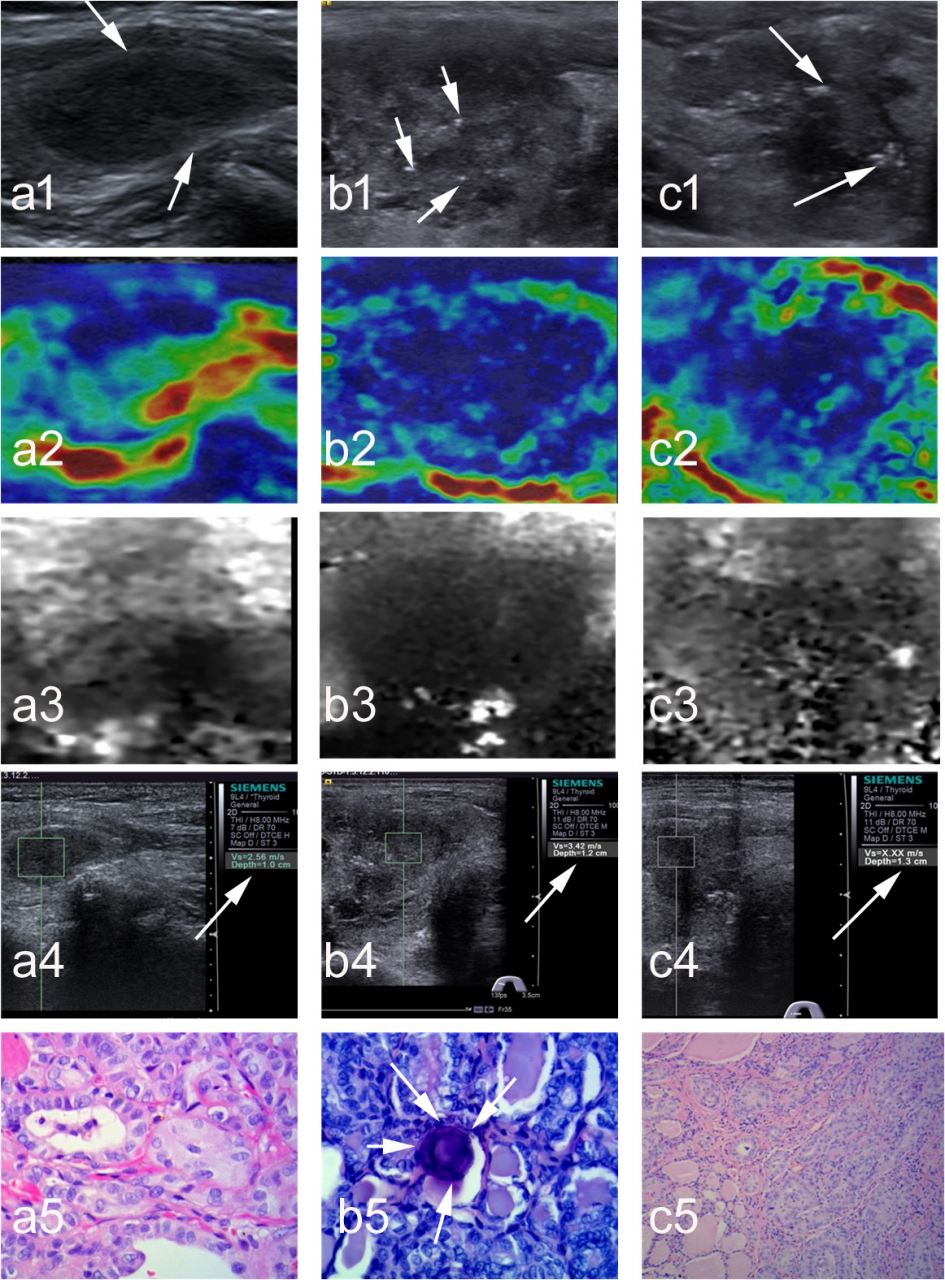
Representative images and histology of non-calcified, micro-calcified and macro-calcified malignant thyroid nodules (a1) At conventional ultrasound, a non-calcified nodule is 13 mm in dimension, solid, hypoechogenic, well defined, wider than tall; (a2-a3)at SE, conventional SE score is 2, and ARFI SE grade is 2; (a4) at SWE, SWS of 2.56 m/s in nodule is assigned (arrows); (a5) Surgery histology confirms a papillary thyroid carcinoma (×400). (b1) At conventional ultrasound, a micro-calcified (arrows) nodule is 22 mm in dimension, solid, hypoechogenic, irregular, wider than tall; (b2-b3) at SE, conventional SE score is 4, and ARFI SE grade is 4; (b4) at SWE, SWS of 3.42 m/s in nodule is assigned (arrow); (b5) Surgery histology confirms a papillary thyroid carcinoma with micro-calcified foci (arrows, ×400). (c1) At conventional ultrasound, a macro-calcified (arrows) nodule is 25 mm in dimension, solid, hypoechogenic, well defined, wider than tall; (c2-c3) at SE, conventional SE score is 3, and ARFI SE grade is 3; (c4) at SWE, SWS of “X.XX m/sec” (i.e., 8.4 m/s) is assigned (arrow); (c5) Surgery histology confirms a papillary thyroid carcinoma (×400).

The distribution of benign vs. malignant nodule, conventional SE score, ARFI SE grade, maximum SWS of nodule, and mean SWS of nodule were significantly different among non-calcification, micro-calcification and macro-calcification groups, whereas patient age, sex, size of nodule, maximum SWS and mean SWS of surrounding tissue were not significantly different. The percentages of malignant nodules in the macro- and micro-calcification groups were 38.1% and 40.9% respectively, compared to 20.9% in the non-calcification group (*p* < 0.001). Both the mean and the maximum SWSs of nodule progressively increased from the non-calcification, to micro-calcification and to macro-calcification groups: the mean SWS values of non-, micro-, and macro-calcified nodules were 2.60 ± 1.49 m/s, 3.27 ± 1.85 m/s, and 3.68 ± 2.26 m/s, respectively (*p* =0.001), and the maximum SWS in these three groups were 3.31 ± 2.01 m/s, 4.64 ± 2.64 m/s, and 4.79 ± 2.67 m/s, respectively (*p*=0.001) (Table 2). This progressive increase of SWS values with the increasing level of calcification was observed in both benign and malignant nodules (Table 3). In contrast to SWS values, the conventional SE scores and ARFI SE grades were not significantly different among the three different calcification nodule groups in either benign or malignant nodules (Table 3). Figures 1 and 2 showed representative images and histology of non-, micro- and macro-calcified benign and malignant nodules, respectively.

**Table 2:** Features of nodules in different calcification groups

Features	Non-calcification group* (n=177)	Micro-calcification group* (n=159)	Macro-calcification group * (n=181)	*P* value @
No. of males/females	34/143	39/120	38/143	0.488
Age #	49.8 ± 13.6 [18-80]	52.5 ± 13.3 [23-82]	52.4 ± 12.0 [23-78]	0.134
No. of nodules	177	159	181	<0.001
No. of benign nodules	140 (79.1)	94 (59.1)	112 (61.9)	
No. of malignant nodules	37 (20.9)	65 (40.9)	69 (38.1)	
Size (mm) #	20.3 ± 10.9 [10.0-62.6]	19.6 ± 8.7 [10.0-59.7]	20.3 ± 8.0 [12.3-53.3]	0.748
Conventional SE score				0.041
No. of score 1	1 (0.6)	0 (0)	0 (0)	
No. of score 2	41 (23.2)	31 (19.4)	40 (22.1)	
No. of score 3	93 (52.5)	64 (40.3)	73 (40.3)	
No. of score 4	35 (19.7)	48 (30.2)	54 (29.8)	
No. of score 5	7 (4.0)	16 (10.1)	14 (7.7)	
ARFI SE grade				0.012
No. of grade 1	1 (0.6)	1 (0.6)	0 ()	
No. of grade 2	41 (23.2)	21 (13.2)	34 (18.0)	
No. of grade 3	89 (50.3)	63 (39.6)	83 (45.9)	
No. of grade 4	34 (19.2)	41 (25.8)	41 (22.7)	
No. of grade 5	10 (5.6)	30 (18.9)	22 (12.2)	
No. of grade 6	2 (1.1)	3 (1.9)	1 (0.6)	
Maximum SWS of nodule # (m/s)	3.31 ± 2.01 [0.56-8.40]	4.64 ± 2.64 [0.70-8.40]	4.79 ± 2.67 [0.54-8.40]	< 0.001
Mean SWS of nodule # (m/s)	2.60 ± 1.49 [0.43-8.21]	3.27 ± 1.85 [0.85-8.01]	3.68 ± 2.26 [0.41-8.32]	< 0.001
Maximum SWS of surrounding tissue # (m/s)	2.55 ± 0.78 [0.91-5.88]	2.60 ± 0.69 [1.26-7.88]	2.65 ± 0.91 [0.82-7.64]	0.466
Mean SWS of surrounding tissue # (m/s)	2.21 ± 0.72 [0.80-5.88]	2.37 ± 0.78 [1.10-6.24]	2.27 ± 0.80 [0.42-6.35]	0.142

* Numbers in parentheses are percentages.

# Data are means ± standard deviations. Ranges are in brackets.

@ *P* values of nonparametric variables were determined by Chi-square test, and *P* values of continuous variables by one way analysis of variation.

Abbreviation: SE, strain elastography; ARFI, acoustic radiation force impulse; SWS, shear-wave speed.

**Table 3:** The elastography features of differently calcified nodules in benign and malignant groups

Features	Benign group			*P* value @	Malignant group			*P* value @
	Non-calcification * (n=140)	Micro-calcification * (n=94)	Macro-calcification * (n=112)		Non-calcification * (n=37)	Micro-calcification * (n=65)	Macro-calcification * (n=69)	
Conventional SE score				0.110				0.242
Score 1	1 (0.7)	0 (0)	0 (0)		0 (0)	0 (0)	0 (0)	
Score 2	40 (28.6)	26 (27.7)	37 (33.0)		1 (2.7)	5 (7.7)	3 (4.3)	
Score 3	88 (62.9)	49 (52.1)	64 (57.1)		5 (13.5)	15 (23.1)	9 (13.0)	
Score 4	9 (6.4)	18 (19.1)	9 (8.0)		26 (70.3)	30 (46.2)	45 (65.2)	
Score 5	2 (1.4)	1 (1.1)	2 (1.8)		5 (13.5)	15 (23.1)	12 (17.4)	
ARFI SE grade				0.036				0.613
Grade 1	1 (0.7)	0 (0)	0 (0)		0 (0)	1 (1.5)	0 (0)	
Grade 2	39 (27.9)	19 (20.2)	31 (27.7)		2 (5.4)	2 (3.1)	3 (4.3)	
Grade 3	86 (61.4)	53 (56.4)	74 (66.1)		3 (8.1)	10 (15.4)	9 (13.0)	
Grade 4	12 (8.6)	15 (16.0)	5 (4.5)		22 (59.5)	26 (40.0)	36 (52.2)	
Grade 5	2 (1.4)	6 (6.4)	2 (1.8)		8 (21.6)	24 (36.9)	20 (29.0)	
Grade 6	0 (0)	1 (1.1)	0 (0)		2 (5.4)	2 (3.1)	1 (1.4)	
Maximum SWS of nodule # (m/s)	2.91 ± 1.74 [0.56-8.40]	3.80 ± 2.39 [1.03-8.21]	3.89± 2.31[0.54-8.40]	<0.001	4.81 ± 2.32 [1.50-8.00]	5.86 ± 2.53 [1.17-8.40]	6.27 ± 2.56 [1.40-8.40]	0.018
Mean SWS of nodule # (m/s)	2.35 ± 1.31 [0.35-8.21]	2.73 ± 1.47 [0.85-8.04]	2.80 ± 1.57[0.41-8.11]	0.029	3.56 ± 1.75 [1.40-8.12]	4.05 ± 2.07 [1.05-8.12]	5.11 ± 2.49 [1.12-8.32]	0.001

* Numbers in parentheses are percentages.

# ata are means ± standard deviations. Ranges are in brackets.

@ *P* values of nonparametric variables were determined by Chi-square test, and *P* values of continuous variables by one way analysis of variation.

Abbreviations: SE, strain elastography; ARFI , acoustic radiation force impulse; SWS, shear-wave speed.

### Cutoff values of elastography for diagnosing malignant nodules in different groups

We then compared the optimal cutoff values of different elastography measurements among the different calcification groups at which the maximal YI, 90% sensitivity or 90% specificity were achieved (Table 4). The cutoff values of SWS of nodule were significantly different among different calcification groups (*p* =0.03), and increased from non-calcification, to micro-calcification, and to macro-calcification groups. Based on the maximum YI, the optimal cutoff values of SWS for diagnosing malignancy in non-, micro-, and macro-calcification nodules were 2.42 m/s, 2.88 m/s, and 3.59 m/s, respectively. At 90% sensitivity, the cutoff values of SWS in the non-, micro-, and macro-calcification groups were 2.38 m/s, 2.71 m/s, and 2.76 m/s respectively, and the corresponding cutoff values to give 90% specificity were 2.67 m/s, 3.24 m/s, and 3.99 m/s, respectively. In contrast, the diagnostic cutoff values of conventional SE score and ARFI SE grade in different calcification groups were not significantly different (*p* = 0.916 and 0.848, respectively).

**Table 4:** Diagnostic cutoff values of conventional SE score, ARFI SE grade, and mean SWS of nodule in the whole and different calcification groups

Methods	The whole group	Calcification groups			P* value
		Non-calcification	Micro-calcification	Macro-calcification	
Conventional SE score					0.916
At the point of maximum YI	>3	>3	>3	>3	
At the point of 90% sensitivity	>2	>2	>2	>2	
At the point of 90% specificity	>3	>3	>4	>3	
ARFI SE grade					0.848
At the point of maximum YI	>3	>3	>3	>3	
At the point of 90% sensitivity	>2	>3	>2	>2	
At the point of 90% specificity	>3	>3	>4	>3	
Mean SWS of nodules (m/s)					0.030
At the point of maximum YI	>2.72	>2.42	>2.88	>3.59	
At the point of 90% sensitivity	>2.61	>2.38	>2.71	>2.76	
At the point of 90% specificity	>3.47	>2.67	>3.24	>3.99	

* Determined using one way analysis of variation in the whole and different calcification groups.

Abbreviations: SE, strain elastography; ARFI , acoustic radiation force impulse; SWS, shear-wave speed; YI, Youden index.

### The diagnostic performances of applying a single uniform or multiple individual SWS cutoff values

If we used individual SWS cutoff points for non- (SWS >2.42 m/s), micro- (SWS >2.88 m/s) and macro-calcification (SWS >3.59 m/s) nodules in the whole group, the AUC was 0.859 (95% CI, 0.826-0.888), significantly better than the AUC of 0.816 (95% CI, 0.780-0.848) if a single uniform cutoff value (SWS >2.72 m/s) was applied to all the nodules regardless of calcification status (*p*=0.011) (Table 5). Applying these distinct SWS cutoff values for different calcification groups, the AUCs in the non-, micro- and macro-calcification groups were 0.906 (95% CI, 0.853-0.945), 0.871 (95% CI, 0.809-0.919) and 0.805 (95% CI, 0.740-0.860) respectively, all higher than corresponding AUCs of 0.799 (95% CI, 0.732-0.855), 0.859 (95% CI, 0.795-0.909) and 0.698 (95% CI, 0.625-0.764) when a single uniform SWS cutoff value (>2.72 m/s) was used. The main effect of applying calcification-specific individual SWS cutoff values in the whole group was specificity, positive predictive value (PPV), and YI, whereas sensitivity and negative predictive value (NPV) was comparable. The specificities in the whole group using multiple individual and single uniform cutoff values were 87.0% and 76.0% respectively; the PPV was 76.3% and 64.2%, and the YI was 0.718 and 0.631, respectively (Table 5). .

**Table 5:** Comparing the diagnostic performances of pSWS for thyroid nodules between a single uniform cutoff value and individual cutoff values

Group	Sensitivity	Specificity	PPV	NPV	YI	ROC	
						AUC*	*P* value#
Non-calcification group							0.004
Single cutoff value (>2.72 m/s)	67.6%	92.1%	69.4%	90.5%	0.597	0.799 (0.732-0.855)	
Individual cutoff value (>2.42 m/s)	91.9%	88.6%	68.0%	97.6%	0.805	0.906 (0.853-0.945)	
Micro-calcification group							0.559
Single cutoff value (>2.72 m/s)	84.6%	87.2%	82.1%	89.1%	0.718	0.859 (0.795-0.909)	
Individual cutoff value (>2.88m/s)	92.3%	81.9%	77.9%	93.9%	0.742	0.871 (0.809-0.919)	
Macro-calcification group							<0.001
Single cutoff value (>2.72 m/s)	91.3%	48.2%	52.1%	90.0%	0.395	0.698 (0.625-0.764)	
Individual cutoff value (>3.59 m/s)	72.5%	86.6%	76.9%	83.6%	0.591	0.805 (0.740-0.860)	
Whole group							0.011
Single cutoff value (>2.72 m/s)	87.1%	76.0%	64.2%	92.3%	0.631	0.816 (0.780-0.848)	
Individual cutoff values@	84.8%	87.0%	76.3%	92.0%	0.718	0.859 (0.826-0.888)	

* Numbers in parentheses are 95% confidence intervals.

# Determined using Z test.

@ SWS of nodule >2.42 m/s, >2.88 m/s and >3.59 m/s were for non-calcified, micro-calcified and macro-calcified nodules in the whole group.

Abbreviations: pSWS, point shear-wave speed; PPV, positive predictive value; NPV, negative predictive value; YI, Youden Index; ROC, receiver operating characteristic; AUROC, area under receiver operating characteristic curve.

## DISCUSSIONS

The main conclusion of our study is that applying multiple individual cutoff values of SWS based on the calcification status of thyroid nodules exhibits better diagnostic performance than using a single cutoff value regardless of calcification status. In our study, the mean SWS increased progressively from non-calcification (2.60 ± 1.49 m/s), to micro-calcification (3.27 ± 1.85 m/s), and to macro-calcification (3.68 ± 2.26 m/s) groups (*p*<0.001). Given that malignant nodules are stiffer than benign nodules [8], we further stratified nodules into malignant and benign groups and observed similar trend of increasing SWS values with higher level of calcification. Consistent with our observation, a previous report on breast lesions showed that highly dense clusters of micro-calcifications and single macro-calcification created the appearance of high SWS, and regional scattered micro-calcifications increased mean SWS by 50% and max SWS by 250% [16]. The strong effect of calcification on SWS values calls for individual cutoff points when diagnosing malignant thyroid nodules using SWE. In contrast, the cutoff values of strain elastography (conventional SE and ARFI SE) in different calcification groups were not significantly different, suggesting that the effect of calcifications on SE was weak. We also observed that the mean SWS of malignant nodules more than doubled that of benign nodules (4.81 vs. 2.29, *p*<0.001, Table 1), confirming the ability of SWE in distinguishing malignant from benign nodules and in diagnosing malignant thyroid nodules. The SWS of surrounding tissues was similar between benign and malignant nodules, indicating the disease specificity of SWS.

YI is a summary measure of sensitivity and specificity and is widely utilized in studies evaluating accuracy of diagnostic tests [25-27]. To achieve maximum YI, the cutoff values of SWS for thyroid nodules were 2.72 m/s, 2.42 m/s, 2.88 m/s, and 3.59 m/s, respectively in the whole, non-calcification, micro-calcification and macro-calcification groups. These values were in line with those reported in previous literatures (range, 2.42-3.39 m/s) [11, 13, 28-31]. In those previous reports, generally a single SWS value was applied for discriminating malignancy in the whole nodules. Our study suggests individual cutoff values of SWS according to varied calcifications in nodules.

This study had a couple of limitations. Firstly, this was a single center, retrospective study. The cutoff values of SWS in different calcification groups warrant future multi-center, prospective validation. Secondly, we did not quantify the level of calcifications in nodules but broadly categorized nodules into three groups.

In conclusion, the mean and maximum SWS of thyroid nodules increases progressively from non-calcification, to micro-calcification and to macro-calcification groups. The diagnostic performance of applying multiple individual cutoff values of pSWS for discriminating malignancy in thyroid nodules is significantly better than that applying a single uniform cutoff value. We recommend individual cutoff values of SWS for diagnosing malignant thyroid nodules with different calcifications.

## MATERIALS AND METHODS

### Patients

All patients were retrospectively identified from patients who came to Shanghai Tenth People's Hospital of Tongji University School of Medicine for thyroid examination. From January 2014 to November 2015. A total of 1,145 consecutive patients with 1,522 thyroid nodules received the examinations of conventional ultrasound, SE and point SWS measurement. Among these patients, 671 patients with 690 nodules met the following enrollment criteria: (1) had fine needle aspiration (FNA) cytology and/or surgery histology within a month after examinations; (2) the size of nodule was ≥10 mm in the greatest dimension; (3) solid nodules at B-mode ultrasound images; and (4) with complete medical information and no surgery treatment performed on the nodules before. Then, 172 nodules were excluded for the following reasons: (1) inadequate cytologic results and without surgery histology (n=42); (2) indeterminable cytologic results and without surgery histology (n=55); (3) being diagnosed as “suspicious for papillary thyroid carcinoma” only at cytologic examination but did not undergo surgery histology (n=23); (4) nodules with annular-like peripheral macro-calcification and/or crescent-like peripheral macro-calcification (n=21); and (5) clustered micro-calcification or macro-calcification foci cannot be completely avoided when measuring the SWS (n=31). Finally, 517 thyroid nodules in 498 patients were included in the analyses, including 346 benign nodules in 330 patients and 171 malignant nodules in 168 patients. Of the 346 benign nodules, 301 nodules were diagnosed by cytology and 45 nodules by surgery histology. Of the 171 malignant nodules, 26 nodules by cytology and 145 nodules by surgery histology (including 135 papillary thyroid carcinomas, 4 follicular thyroid carcinomas, 3 medullary carcinomas and 2 anaplastic carcinomas). This study was approved by the Ethical Committee of the Shanghai Tenth People's Hospital of Tongji University School of Medicine, and the requirement to obtain informed consent was waived.

### Imaging procedures

B-mode ultrasound, SE and point SWS measurement for thyroid were performed with the same S2000 ultrasound instrument (Siemens Medical Solutions, Mountain View, CA, USA). A 9L4-linear transducer (frequency range, 4-9 MHz) was used for thyroid examination. The patients were placed in a supine position with dorsal flexion of the neck. The gain, focus position and depth of instrument were adjusted appropriately to ensure that the nodules displayed completely and conspicuously on the screen. Detailed procedures of examination were described previously [11].

### Image Interpretation

All the images of B-mode ultrasound, SE, and point SWS measurement were analyzed in the same setting and in a blind manner by two experienced thyroid radiologists (B.J.L. and X.L.L.) .The identification of patients, clinical results and pathology results were anonymous to the investigators. In case of disagreement in the evaluation between the two radiologists, a third senior radiologist (B.D.C.) reviewed the images to make the final decision.

At B-mode ultrasound, the size of nodule was measured in longitudinal and transverse planes, and the largest dimension was used to measure the size of nodule. The other interpreted features of the nodule included: calcification (non-, micro-, or macro-calcification), echogenicity (hyper-, iso-, or hypo-echogenicity, and marked hypoechogenicity), shape (taller-than-wide or wider-than-tall), and margin (irregular, microlobulated, or well-defined). If a thyroid nodule had a combination of micro-calcification and macro-calcification, the nodule was classified as a macro-calcification nodule.

The conventional SE score of nodules was classified with a five-score system: score 1, the entire nodule is soft; score 2, part of nodule is hard; score 3, only margin of nodule is soft; score 4, the entire nodule is hard; and score 5, the entire nodule and surrounding area are hard, according to the most recent guidelines and recommendations for clinical use of elastography by the World Federation for Ultrasound in Medicine and Biology [9]. Acoustic radiation force impulse (ARFI) SE grade for the thyroid nodules was divided into grade 1 to 6: grade 1, predominantly white; grade 2, predominantly white with few black portions; grade 3, black and white portion equally; grade 4, predominantly black with a few white spots; grade 5, almost completely black, and grade 6, completely dark, as we recently described [11].

For point SWS measurement, among 7 measured values of SWS for nodule and surrounding tissue, the maximum value of SWS was used as the first parameter. Then, the maximum and minimum values were eliminated, and the average of remaining 5 values was used as the second parameter of SWS. Previous reports have shown that five measurements were sufficient to assess thyroid stiffness [32, 33]. According to the manufacturer's suggestion and relevant reports [11, 34-36], the measurement result of “X.XX m/sec” was replaced by 0 m/s or 8.4 m/s, with 0 m/s corresponding to the cystic portion and 8.4 m/s corresponding to the solid portion. The SWS ranged from 0.35 m/s to 8.40 m/s in this study.

### Statistical Analyses

All the statistical analyses were performed with SPSS software (version 22.0; SPSS, Chicago, Ill) and MedCalc software (version 13.0; MedCalc,). Quantitative values were expressed as means ± standard deviations (SD) and ranges. Nonparametric variables were analyzed by the Chi-square test, and continuous variables by independent t test or one-way analysis of variance (ANOVA). The single and individual cutoff values of conventional SE score, ARFI SE grade and SWS were calculated by ROC curve yielding the maximum Youden index (YI) (i.e., sensitivity + specificity - 1), 90% sensitivity and 90% specificity [25, 27]. The differences of cutoff values among different groups were tested by one-way ANOVA. The sensitivity, specificity, positive predictive value (PPV), negative predictive value (NPV) and YI were calculated applying the diagnostic 2×2 contingency tables. The diagnostic performances between single and individual cutoff values for thyroid nodules were compared by Z test. A two-tailed P value <0.05 was considered statistically significant.
